# Contaminated Airway Task Training: How to Adapt an Existing Airway Manikin Head for Active Airway Soiling

**DOI:** 10.7759/cureus.51285

**Published:** 2023-12-29

**Authors:** Melissa Bouwsema, Amar Chakraborty, Akshay Rajaram, Loren Fleming, Adam Parks

**Affiliations:** 1 Emergency Medicine, Queen's University, Kingston, CAN; 2 Emergency Medicine, Massachusetts General Hospital, Boston, USA; 3 Clinical Simulation Centre, Queen's University, Kingston, CAN; 4 Emergency Medicine, Dalhousie University, Halifax, CAN

**Keywords:** prehospital and retrieval medicine, emergency medicine, task-trainer, simulation, medical education, suction assisted laryngoscopy airway decontamination (salad), contaminated airway, advanced airway management

## Abstract

The massively contaminated airway is an important and often daunting entity for airway providers. Although massively contaminated airways are considered high acuity, low-occurrence presentations in emergency medicine and pre-hospital settings, formal training in the management of contaminated airways is heterogeneous and infrequent. To facilitate training and augment simulation, an airway task trainer is critical.

To our knowledge, this is the first readily accessible, peer-reviewed, detailed technical report to build a low-cost, high-fidelity, contaminated airway task trainer. This trainer can be seamlessly integrated into simulated resuscitation scenarios and/or airway training workshops, reinforcing skill acquisition and retention for the management of the massively contaminated airway.

## Introduction

Intubation remains a high-risk procedure where first-pass success is critical, as mortality and morbidity increase with multiple attempts [1]. Within the arena of the airway, many factors can reduce the first-pass success rate, including the presence of contaminants like blood and vomit [2].

Although the incidence of a massively contaminated airway is not well known, the contaminated airway is not a rare occurrence, with recent data suggesting that 20-30% of out-of-hospital cardiac arrests involve some degree of airway soiling [3,4]. Accordingly, providers must be well-rehearsed in the management of the contaminated airway, as clinical encounters alone cannot be relied upon to effectively prepare for these potentially catastrophic scenarios and their associated sequelae (e.g., aspiration, hypoxia, cardiac arrest). Anecdotally, contaminated airways receive less attention in training compared to rarer “can’t-intubate-can’t-oxygenate” events. The latter has an incidence of approximately 1 in 50,000 [5], in contrast to contaminated airway events, which have an incidence of 1 in 300 to 1 in 10,000 [6].

The College of Family Physicians of Canada (CFPC) and Royal College of Physicians and Surgeons of Canada (RCPSC) emergency medicine (EM) residency training objectives do not explicitly discuss management of the contaminated airway, furthering the ambiguity of training requirements and allowing for this heterogeneity [7,8]. As part of a needs assessment, prior to the publication of this technical report, we surveyed Canadian EM residency program directors. Of those that responded, only 4 out of 12 (33%) RCPSC EM programs and 3 out of 6 (50%) CFPC EM programs offer structured, formal curricula in contaminated airway management.

Task trainers are an effective method to train procedural skills, with evidence to show improvement in technical skill acquisition and retention [9]. These trainers can be used for targeted technical skill training and as an adjunct in simulation scenarios. Physical and conceptual fidelity are important prerequisites for task trainers’ effectiveness, but achieving high degrees of fidelity with commercially designed task trainers can be challenging and expensive [9,10]. Modifications to existing task trainers can balance the efficient use of resources with the need for fidelity.

Our report describes an accessible blueprint for building a contaminated airway task trainer, highlighting its fidelity, low cost, and practicality.

## Technical report

We modified a Laerdal Airway Management Trainer owned by our local Clinical Simulation Centre for conventional airway training without affecting the basic function of the manikin. Our refined final design is presented below, with the rationale for specific design choices outlined. This design can be applied to most airway trainer heads that have an esophageal port (e.g., the Gaumard HAL Airway and cardiopulmonary resuscitation trainer).

The basic design for the adapted manikin head involves removing the stomach attachment and replacing it with a pump system to force “contaminant” through the esophagus. These changes facilitate the simulation of a grossly contaminated airway (e.g., massive upper gastrointestinal bleed). As part of the design, we plugged the lungs to prevent contaminants from draining into these reservoirs, which negatively impact the priming of the system and are challenging to clean.

The materials needed for replication of this design can be found in Table 1. Item numbers specific to the store we purchased from have been included to allow ease of finding exact materials.

**Table 1:** Components and price list PVC: Polyvinyl chloride; MPT: Male pipe threads; FHT: Female hose threads; SAE: Standard American English/American type hose clamp; GPH: Gallons per hour

Start-up cost: task trainer modifications			
	Item	Cost (CAN $)	Comments
A	Laerdal Airway Management Trainer (1) - Laerdal Medical #2500033	$3,299 + tax	
B	Laerdal Airway Management Trainer, storage container	Included in above	
C	Laerdal Airway Management Trainer, storage container hinge mechanism	Included in above	This is used to stiffen the rubber hose (H) going into the contaminant.
D	Rubber beverage stoppers - Rabbit Wine and Beverage Bottle Stoppers (assorted colors, set of four) - https://www.amazon.ca/Rabbit-Beverage-Bottle-Stoppers-Assorted/dp/B005N0WCE4/ref=sr_1_11?keywords=wine+stoppers+for+wine+bottles&qid=1684690679&sprefix=wine+stopp%2Caps%2C130&sr=8-11	$18.55 + tax	These are for the lungs to prevent contaminants from getting into them. If the lungs are left open, this leads to cleaning challenges, as well as inhibition of priming of the system.
E	Teflon tape (2) - Lowes #282322	$4.58 + tax	This is one of the most important purchases for leak prevention. Use it anytime you see threading in the system.
F	¾-3” PVC Sch 80 Nipple (MPT) (2) - Lowes #2887386	$5.78 + tax	Used to connect set up to manikin.
G	¾-1 ½” stainless steel hose clamp SAE #16 (1) - Lowes #3311711	$10.49 + tax	Only need two clamps out of the pack of 10.
H	Steel washing machine hose (1), ¾” FHT x ¾” FHT connections, 72” (6-ft) hose length - Lowes #2807218	$16.99 + tax	Braided stainless steel washing machine hoses are both stronger, last longer, and don’t kink. Rubber hoses are more cost effective up front, but will need more frequent maintenance/replacement. This hose is used for the manikin-to-pump connection.
I	Zip ties, 20/bag - Lowes #105499	$4.99 + tax	
J	Drill pump (1), Male ends, Maximum flow rate of 205 GPH (776 L/h) - Canadian Tire #062-3531-0	$39.99 + tax	Labeling the directions on the pump as tank and head allows for easier disassembly and subsequent reassembly. May be used with any high-speed drill that is 1200 RPM or higher.
K	Rubber washing machine hose (1),¾” FHT x ¾” FHT connections, 72” (6-ft) hose length - Lowes #2807213	$19.99 + tax	Rubber hoses will also work for this design but will get brittle with age, and will need to be replaced more frequently. This hose is used for the pump-to-fluid reservoir connection.
L	20L reservoir, Plastic carboy with a cap - Canadian Tire 085-4062–6	$25.99 + tax	The ideal reservoir would be a clear or translucent container to make it easier to see the fluid tank level. We only had an opaque carboy available at the local hardware store. Can alternatively use a large bucket which allows for ongoing monitoring of contaminant levels.
M	Variable speed corded drill ⅜” - Lowes #1046152	$79.99 + tax	Need a more powerful drill the longer the washing machine tubing is. More power/higher amperage equates to both a better lifespan of the drill, as well as more contaminant coming out.
	Total with Manikin Head	$3,526 + tax	
	Total without Manikin Head	$227.34 + tax	
	Recurring Costs (Contaminant Ingredients)		
N	Balloons - Dollarama	$1.25 + tax	Only necessary if removal of the contamination set up, to allow use of the manikin head for conventional airway practice. The balloon replaces the stomach component.
O	Laerdal blood-colored concentrate - Laerdal Medical #300-00750	$35.00 + tax	
P	Dish soap - Dollarama	$2.75 + tax	
Q	Xanthan gum - Bulk barn #011584	$32.76 + tax	One bag of powder will make ~ 6 “batches” of simulated vomit (depending on consistency).

Assembly

Note that all the connections only need to be hand-tight.

Step 1: Disassemble the Laerdal Airway Management Trainer Storage Container (Table 1B). To do this, remove the metal piece holding the hinge mechanism, and save for step 14. Separate the container into top and bottom sections.

Step 2: Place the Laerdal Airway Management Trainer (Table 1A). into the bottom half of the storage container (Table 1B) This storage container is watertight, and so allows for any excess “contaminant” to be collected without spilling onto the floor.

**Figure 1:**
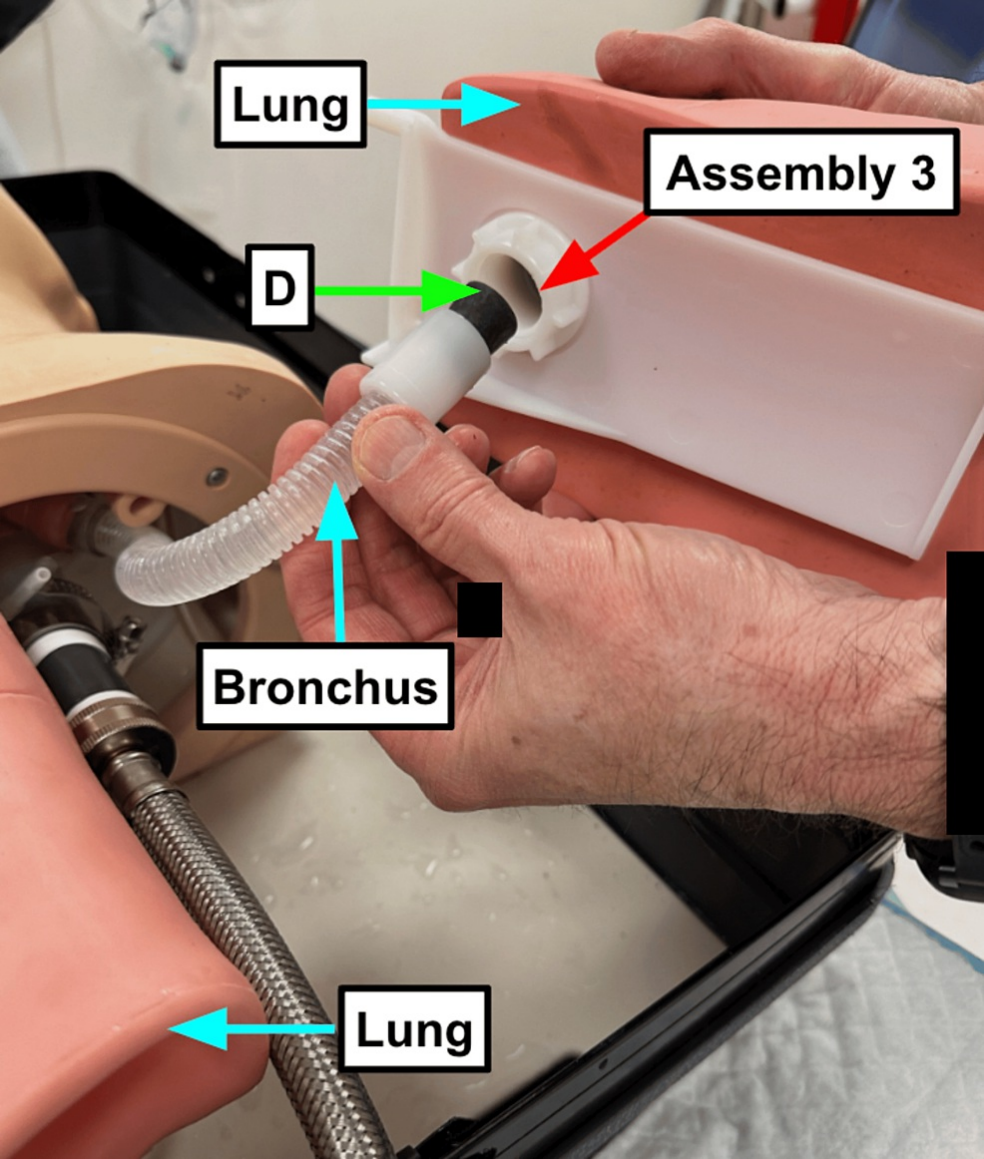
Beverage stoppers prevent contaminants from leaking into the lungs Blue arrow: Existing manikin component Green arrow: Component referenced in Table 1 Red arrow: Step referenced in the manuscript

Step 3: Remove the lungs, and place rubber beverage stoppers (Table 1D) into the bronchi tubing connections (Figure 1).

Step 4: Reattach the lungs to the bronchi attachment.

Step 5: Cut off the esophagus attachment for the stomach on the Laerdal Airway Management Trainer (Table 1A).

**Figure 2:**
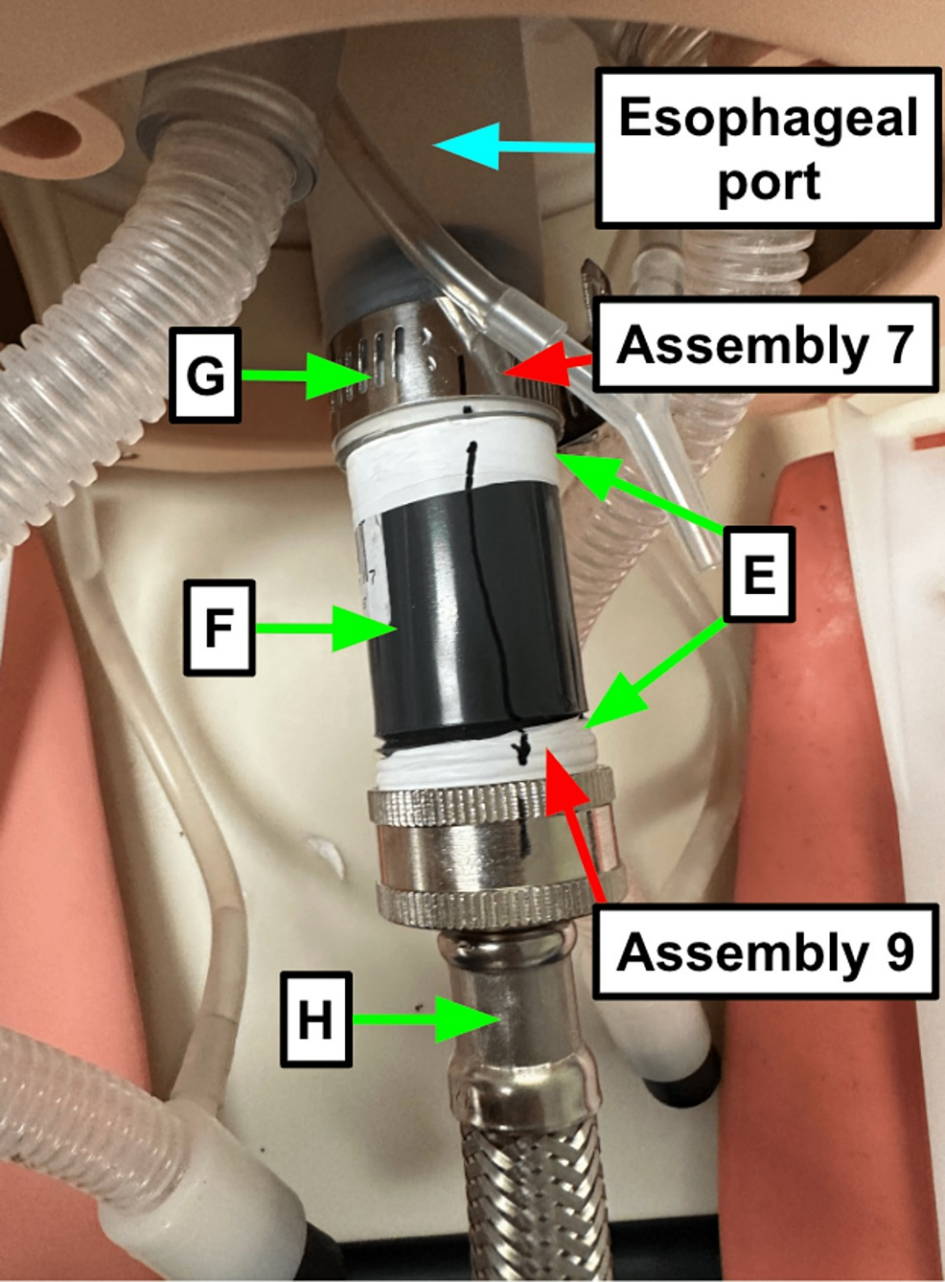
A hose clamp secures the PVC nipple into the esophageal port Note the marking to indicate the alignment of connections; if the line is disrupted, the connections need to be tightened. Blue arrow: Existing manikin component Green arrow: Component referenced in Table 1 Red arrow: Step referenced in the manuscript

Step 6: Wrap Teflon tape (Table 1E) around both ends of the 3” long polyvinyl chloride (PVC) nipple (Table 1F, Figure 2).

Step 7: Place the 3” PVC nipple (Table 1F) into the esophageal port of the manikin head and secure in place with the hose clamp (Table 1G). This holds the system into the esophageal port without any leaks of the contaminant. Use a screwdriver to tighten the clamp until you get resistance. This is not a high-pressure system so it needs to be “secure” but not immovable (Figure 2).

Step 8: Attach the stainless steel washer hose (Table 1H) to the free end of the 3” PVC nipple (Table 1F, Figure 2).

Step 9: Use a marking pen to create a visual cue of the connections being aligned (Figure 2).

**Figure 3:**
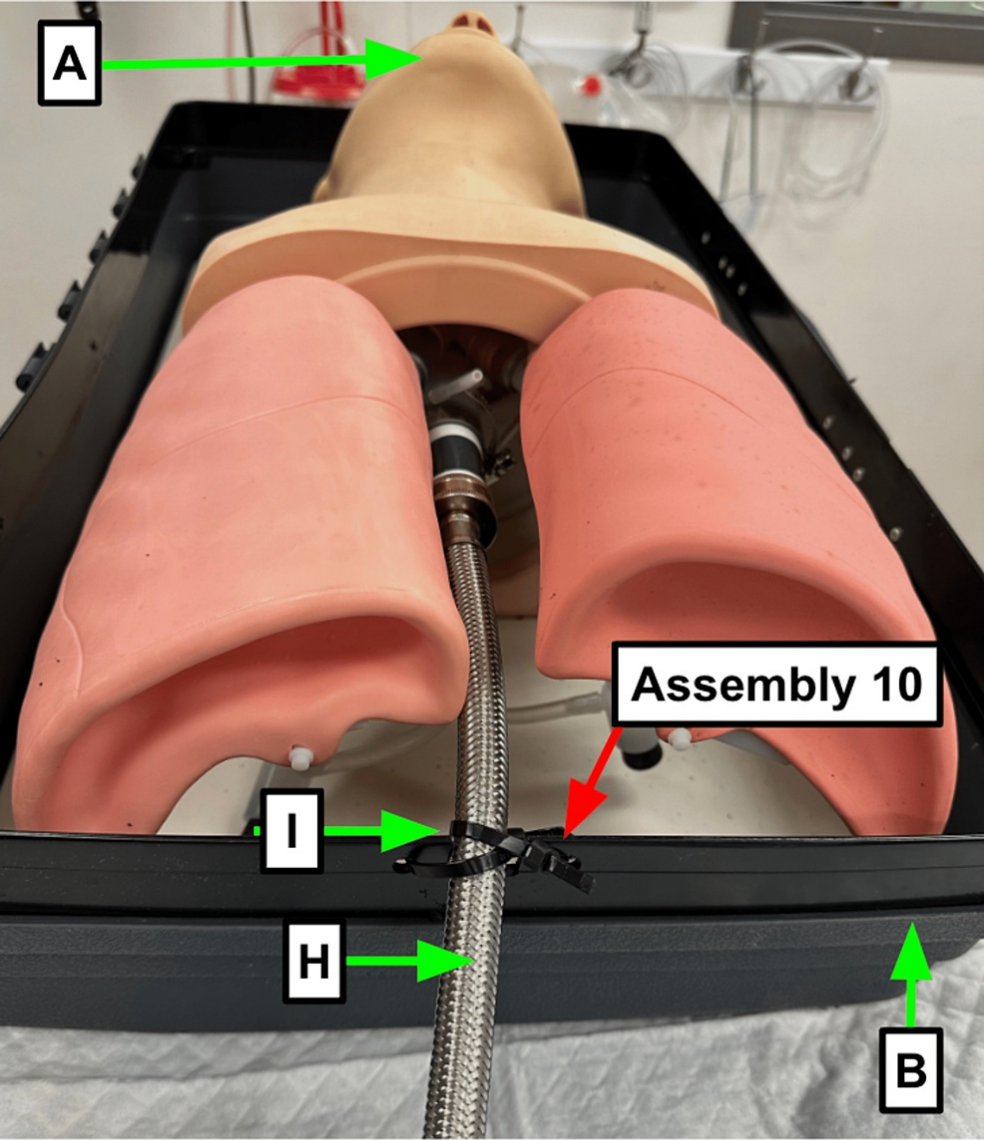
Zip ties hold the stainless steel washing machine hose against the container and reduce spinning and kinking Blue arrow: Existing manikin component Green arrow: Component referenced in Table 1 Red arrow: Step referenced in the manuscript

Step 10: Secure the stainless steel washer hose (Table 1H) to the bottom half of the storage container (Table 1B) using zip ties (Table 1I, Figure 3).

**Figure 4:**
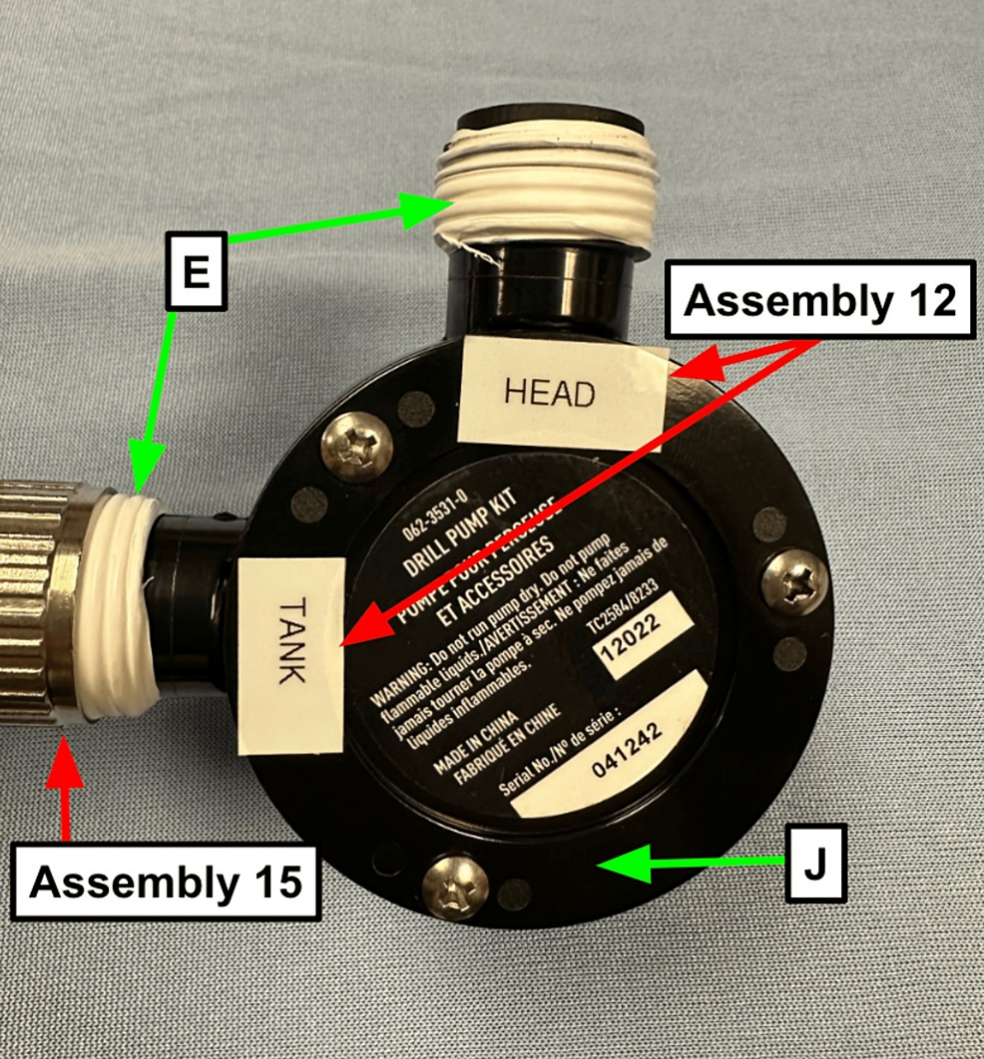
Labeled drill pump Note the White Teflon tape (Table 1E) wrapped around the connection ports. Blue arrow: Existing manikin component Green arrow: Component referenced in Table 1 Red arrow: Step referenced in the manuscript

Step 11: Wrap Teflon tape (Table 1E) around threaded ports on the drill pump (Table 1J, Figure 4).

Step 12: Label the ports on your drill pump (Table 1J). The head end should always connect to the hose going to the manikin head (Table 1H), and the tank end should connect to the hose pulling the contaminant (Table 1K, Figure 4).

**Figure 5:**
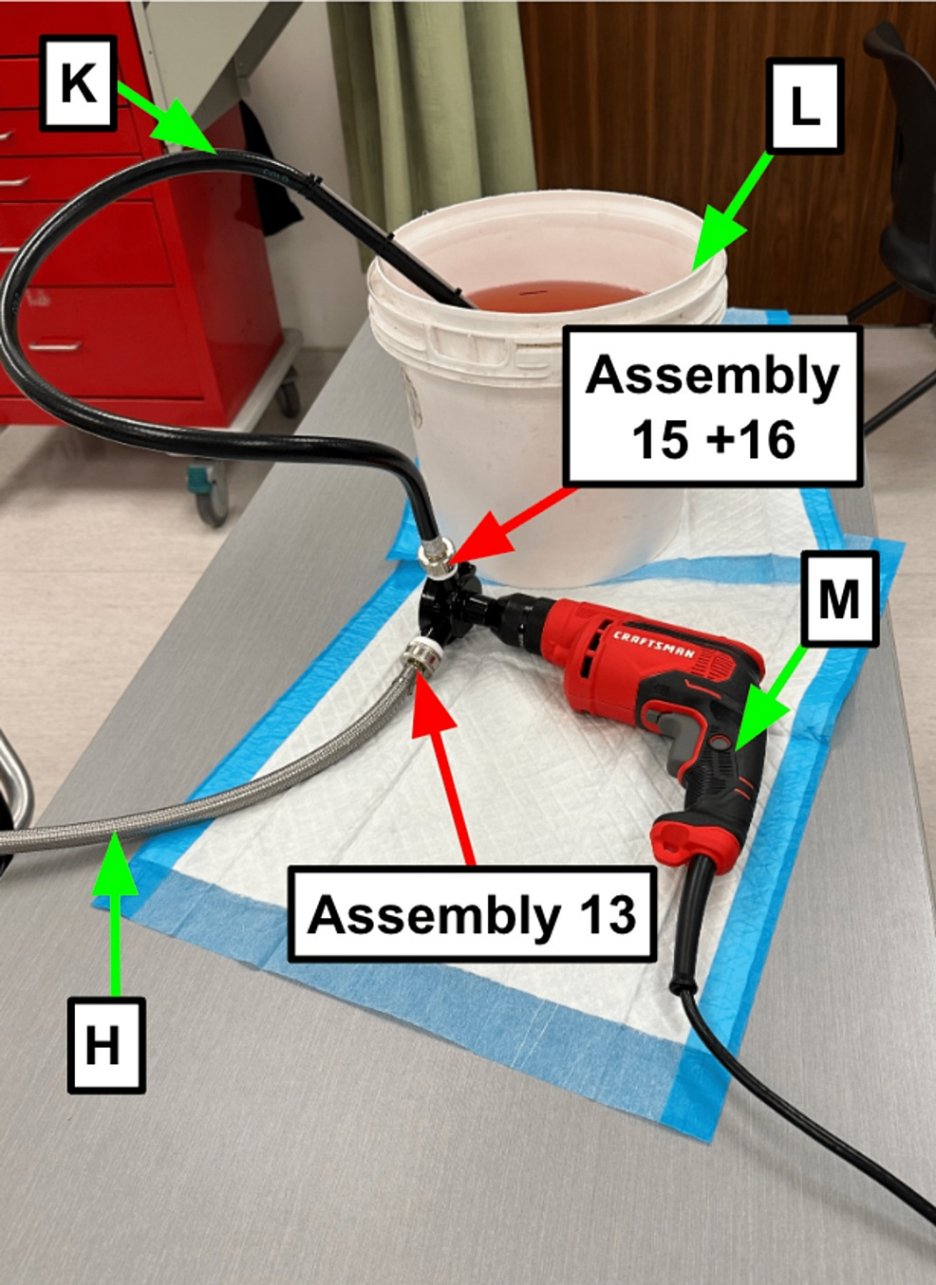
Configuration of the drill to pump, and hoses running to contaminant reservoir and manikin head Blue arrow: Existing manikin component Green arrow: Component referenced in Table 1 Red arrow: Step referenced in the manuscript

Step 13: Attach the free end of the stainless steel washer hose coming from the manikin head (Table 1H) to the drill pump (Table 1J, Figure 5).

**Figure 6:**
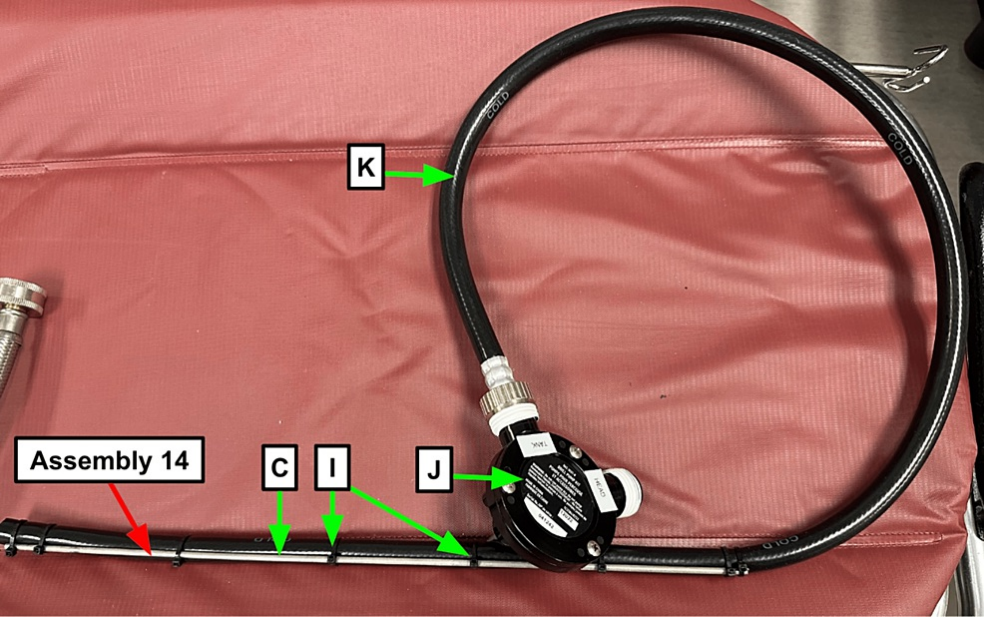
Metal rod zip tied to the hose to create structure and therefore allow the hose to be guided to the contaminant more easily Blue arrow: Existing manikin component Green arrow: Component referenced in Table 1 Red arrow: Step referenced in the manuscript

Step 14: Take the rubber hose (Table 1K), and cut it to the necessary length to reach your fill tank. Secure the metal piece from the hinge mechanism of the storage container (Table 1C) referenced in step 1 to the distal end with zip ties to create a structure for directing it into the contaminant (Figure 6).

Step 15: Secure the female end of the rubber hose (Table 1K) to the drill pump (Table 1J, Figure 5).

Step 16: Remove the lid of the contaminant reservoir (Table 1L) and direct the structured end of the rubber hose (Table 1K) into your contaminant (Figure 5).

**Figure 7:**
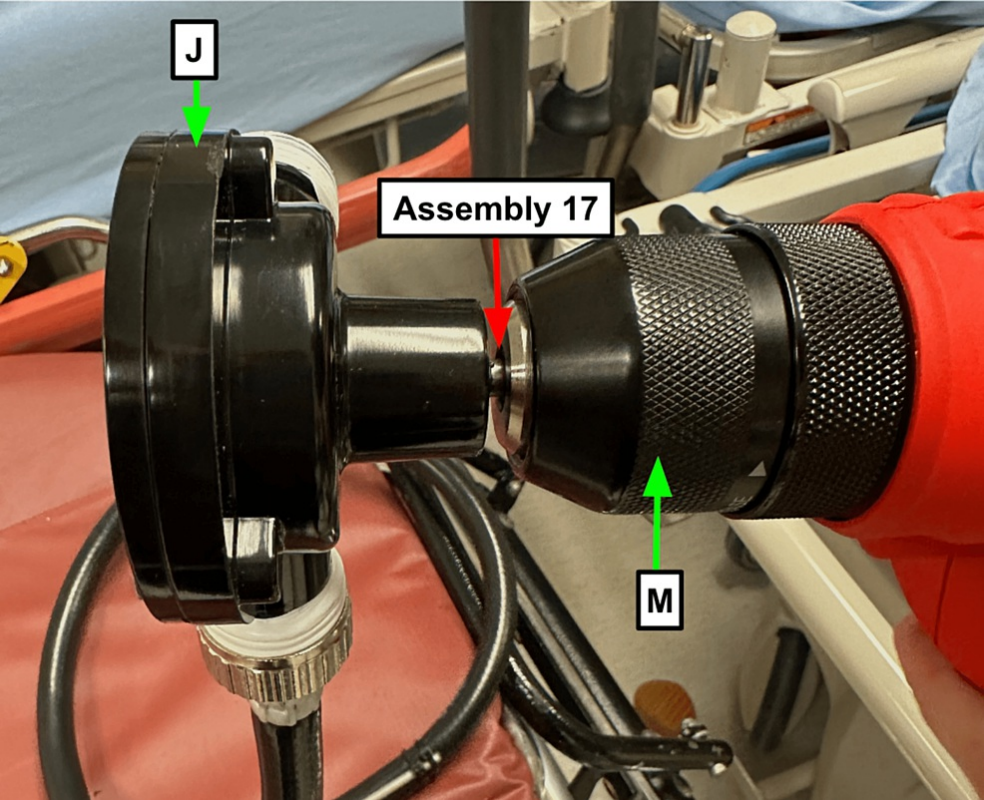
Leave a space between the drill and the pump so it doesn’t overheat Blue arrow: Existing manikin component Green arrow: Component referenced in Table 1 Red arrow: Step referenced in the manuscript

Step 17: Secure the pump (Table 1J) into the chuck of the drill (Table 1M) and leave a few millimeters gap here to prevent overheating when the drill is in use (Figure 7).

**Figure 8:**
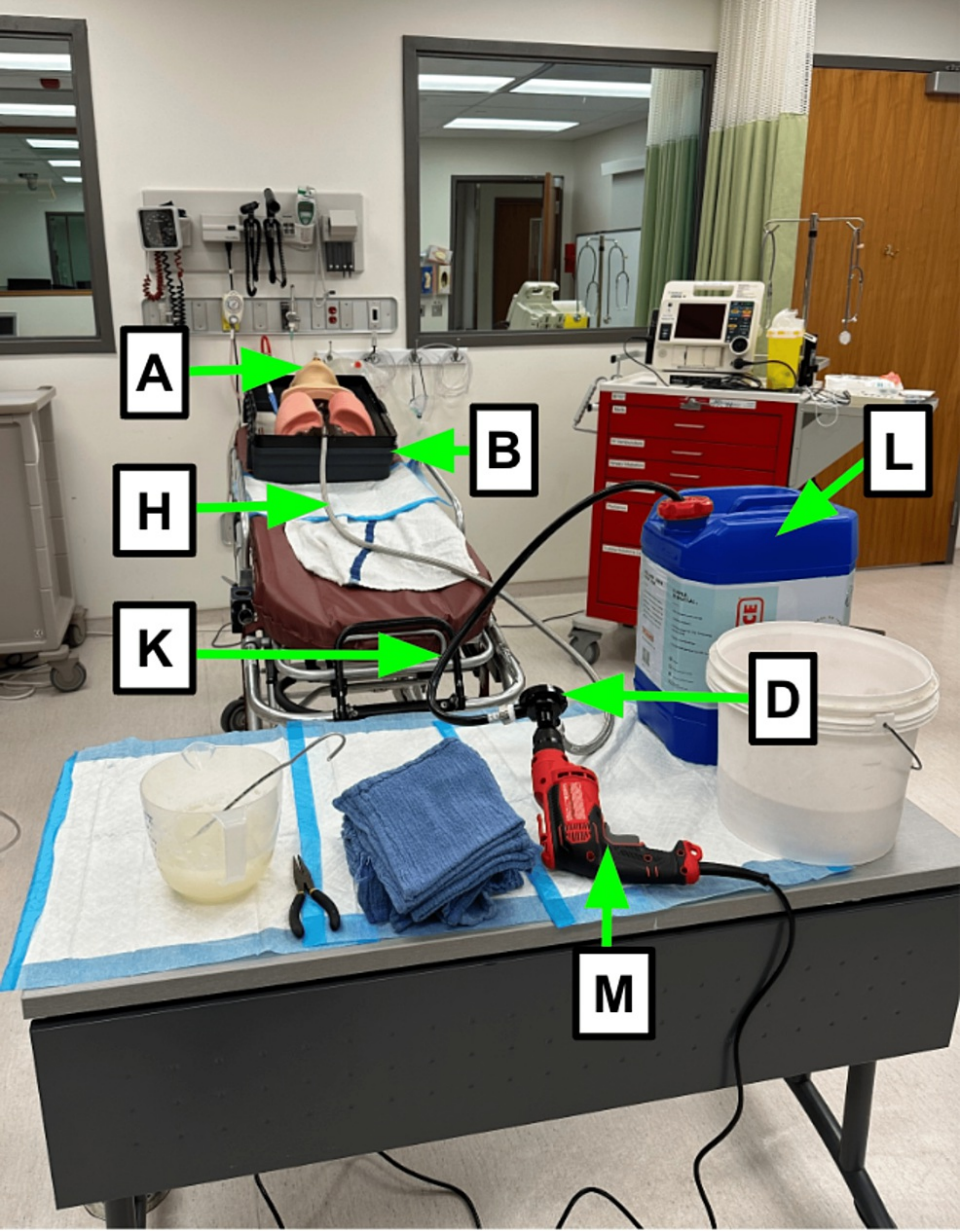
Completed setup Blue arrow: Existing manikin component Green arrow: Component referenced in Table 1 Red arrow: Step referenced in the manuscript

Step 18: Final check of the entire system (Figure 8).

Operation

Note 1: A clear carboy or large bucket is ideal for the contaminant reservoir - this allows for the operator to see how much contaminant has been used, and refill as needed.

Note 2: Select a recipe for your contaminant from Table 2 and prepare accordingly.

**Table 2:** Contaminant recipes PPE: Personal protective equipment; CHF: Congestive heart failure

	Type	Recipe	Rationale
A	Water only	6L water	For beginner trainees. Allows for technical practice of managing contaminants in the airway and active suctioning techniques. Does not obstruct the view of the airway completely/trainees will still be able to visualize the airway through contaminant.
B	Hemoptysis/hematemesis	6L water ≥ 20mL Laerdal blood-colored concentrate. Stir to integrate. Titrate to the desired color.	This allows for enough “color” that the view of the airway is obstructed. The blood concentrate > food coloring creates a foaming effect, similar to pulmonary edema but to a less dramatic extent than recipe C. Note that this mixture will stain - have the participants wear gowns/PPE accordingly. The advantage of this contaminant is that it doesn’t contain organic material - the system can be rinsed after use, for simplified cleaning/take down of the system.
C	Pulmonary edema	6L warm water 1mL dish soap. Mix with a whisk to integrate some air into the mixture before adding to the system. Warm water enhances the foaming effect and will be more dramatic than if using cold or room temperature water.	Run through the system with some air - this will enhance foam and create a pulmonary edema-like effect. Select training around severe CHF or post-drowning airway management. The advantage of this contaminant is that it doesn’t contain organic material - the system can be rinsed after use, for simplified cleaning/take down of the system.
D	Gastric contents	6L of water 7.5 tbsp xanthan gum powder. Use a blender and mix gradually. This will thicken as it stands - may need to gradually introduce more water depending on the length of the session/if the mixture is prepared the night before. The more viscous mixture more closely resembles gastric contents.	For advanced trainees. This recipe will add the complexity of managing the volume of contaminant, as well as different viscosities. Xanthan gum is organic - to be cleaned/prevent molding, a bleach mixture is needed to clean (which will easily double your clean-up time). The residue is also hard to remove and needs to be scrubbed/rinsed repeatedly.

Note 3: Place the carboy at the same height as the manikin, this requires less drill demand to prime and run the system (Figure 8, Video 1).

**Video 1:** Operating the system - note the reservoir is at approximately the same height as the manikin head

Note 4: Ensure the drill direction is set to “forward.”

Note 5: The contaminant doesn’t flow if the drill isn’t running, and the line needs to be primed. The operator runs the drill until the contaminant flows up through the manikin’s mouth, and then reduces pressure on the drill trigger to maintain a continuous volume of contaminant flowing into the oropharynx (Video 2).

**Video 2:** Suction-assisted laryngoscopy airway decontamination (SALAD) practice with continuous airway soiling by the task trainer

Note 6: A tip for fidelity - take manikin lubricant and spray it around the mouth/on the suction device. This will make everything slippery, and more closely resemble the way things become hard to grasp when there is contaminant spilling up through the airway.

Disassembly

Step 1: Suction any excess contaminant that’s leaked from the mouth into the Laerdal Airway Management Trainer - Storage Container, and from the manikin (Table 1B, Video 3).

**Video 3:** Suction any excess contaminant from the container and the manikin

Step 2: Reverse the drill and pump the remaining contaminant back out of the manikin (Table 1A).

Step 3: Empty any remaining contaminant solution into the sink or toilet and rinse.

Step 4: Disconnect lungs from your airway simulator, and clean and dry them separately.

Step 5: Release the zip tie holding the stainless steel hose to the base - remove the head from the base and place it in the sink.

Step 6: Refill your carboy with hot water and bleach solution (2 tbsp/4L) if using gastric contents recipe (Table 2D), or just hot water if using hemoptysis/hematemesis or pulmonary edema recipes (Tables 2B, 2C) - pump through the manikin until everything that runs out is clear.

Step 7: Carefully disconnect and drain hoses.

Step 8: Allow the manikin head (Table 1A) to drain, and dry with paper towels.

**Figure 9:**
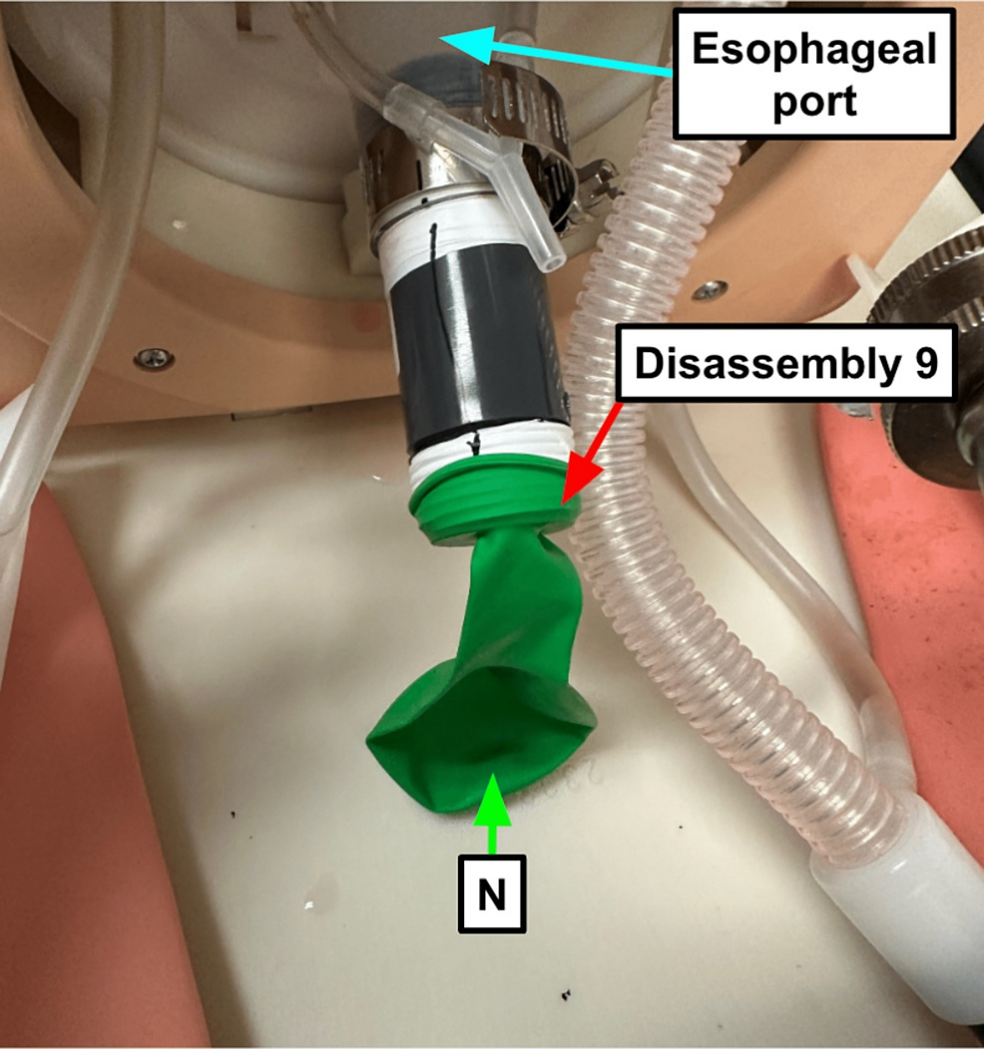
A balloon over the esophageal port allows you to remove the contamination setup and use the manikin head for conventional airway practice Blue arrow: Existing manikin component Green arrow: Component referenced in Table 1 Red arrow: Step referenced in the manuscript

Step 9: Secure a balloon (Table 1N) over the esophageal port to replace the stomach if using the manikin for conventional airway training (Figure 9).

Step 10: Store as needed. With the manikin head separated from the contamination setup, the head can be replaced in its storage box. The contamination setup can be stored inside the storage box from a space perspective, aside from the carboy/contaminant reservoir.

Other considerations for delivering a task trainer session

Note 1: A sink in the room will greatly enhance your clean-up.

Note 2: Have many towels available as this is an inherently messy training session.

Note 3: Electrical safety is important. Ensure the drill is kept dry throughout the simulation. The manikin and contaminant pump setup should be positioned on a non-electrical bed or surface. The use of surge protectors for drill electrical connections is recommended.

Note 4: If running a simulation session rather than task practice - begin the scenario with the adult simulation manikin set up for initial assessment and resuscitation including vitals, IV insertion, and physician examination, and then move to the modified task trainer for skill practice.

Note 5: Have either wall or portable suction with additional suction drainage containers available. A shop vacuum may also be outfitted with ventilator/suction tubing and waterproof tape. This allows for continuous suctioning while minimizing suction canister changeover, although may decrease overall fidelity.

Note 6: Select your contaminant according to the trainees participating in the session, and for the specific clinical presentation you are training. Options are outlined in Table 2.

## Discussion

The contaminated airway is an important and not uncommon scenario encountered in resuscitation. Airway decontamination in these situations is critical, yet opportunities to practice methods of control (e.g., suction-assisted laryngoscopy airway decontamination (SALAD), suction-assisted airway catheter insertion, esophageal diversion) are limited. Simulation is the ideal environment for trainees to prepare, yet conventional manikins are incapable of achieving physical and conceptual fidelity. We modified a manikin head using parts easily available at local hardware stores to create a contaminated airway task trainer.

By modifying a previously available blueprint, we were able to build the trainer in under five hours. Of note, this design can be applied to any airway trainer that has a distinct esophageal port. The cost including a new manikin head is just under $4,000. When the manikin head is already owned, the cost is approximately $250 CAD (see Table 1 for cost breakdown), reducing the cost burden. The manikin used in our design had been decommissioned from regular simulation training due to some minor damage to the mouth and tracheal components of the manikin head, allowing us to trial various designs. Any damaged manikin head that is no longer usable for conventional airway training can be repurposed for this process.

The trainer is easy to use and transport and therefore may be employed to train teams operating in different settings (e.g., pre-hospital, emergency department, critical care unit, operating room) to further enhance the fidelity of contaminated airway simulations.

The trainer holds significant volumes of contaminant, can simulate active contamination, and is amenable to different contaminant viscosity (see suggested recipes outlined in Table 2). For example, in a test of the trainer with our authorship team, we simulated bloody emesis with readily available blood-colored concentrate mixed with water. This produced a foaming effect and adequately blurred the camera and visualization of the vocal cords on the video laryngoscope. 

DuCanto et al. first described a novel airway training tool to simulate vomiting and introduced the SALAD approach, but provided only a brief outline of the tool’s assembly [11]. Their system cost approximately $3200 CAD and also modified an airway manikin head. In contrast, we used a decommissioned Laerdal Airway Management Trainer based on other adaptations [12]. In addition to the cost savings associated with using an existing airway management trainer, the contaminant attachment may be removed allowing the airway trainer to be used conventionally. 

Limitations

While our trainer was assembled in approximately five hours, an additional 10-12 hours were invested in research, procurement of supplies, experimentation, and revisions to the design. We recognize that some settings may not have access to simulation technicians who undertook the majority of this work and have extensive knowledge in designing and creating task trainers. For someone with less technical experience, more assembly time may be needed. 

In contrast to the DuCanto system, the use of this task trainer requires two operators: one to manage the drill pump and another to manage the simulation scenario and/or instruction.

If using a contaminant with organic material, the system needs to be robustly cleaned with bleach or vinegar. This process will drastically increase cleaning time. This type of cleaning will also degrade some components faster (e.g., drill pump), creating maintenance costs. While there is enhanced fidelity to the viscosity of using organic materials as the contaminant, we balance this aim with the learning objectives of the target audience for a given training session (i.e., previous experience with contaminated airways and mastery of fundamental approach). The drill pump also limits the use of particulate matter (e.g., simulated partially digested food, simulated blood clots) in soiled airway simulation; however, if this fidelity is required, the hose proximal to the drill pump (Table 1B) can be pre-loaded with this simulated particulate.

Next steps

Our objective was to construct a task trainer for use in simulations addressing contaminated airway management. Our institutions have robust airway management curricula targeting trainees from various disciplines. Moving forward, we plan to deploy the trainer in these curricula to help teach and develop an approach to the contaminated airway as well as collect feedback from users regarding the trainer. 

## Conclusions

Contaminated airways are common and challenging to manage. Effective management of the soiled airway is a technical skill that any resuscitationist must master. Simulation is an effective tool to train technical skills, and we have outlined blueprints for a cost-effective contaminated airway task trainer that can be easily replicated. Furthermore, this high-fidelity task trainer also allows for expanded applications of an existing airway manikin head, as the modifications outlined do not inhibit the use of the manikin head for conventional airway training. This modified task trainer can be easily integrated into existing airway curricula, can be used by a variety of disciplines and providers, and can be used for both dedicated technical skills training and resuscitation simulation scenarios.
